# Genetic, phytochemical and morphological identification and genetic diversity of selected *Moringa* species

**DOI:** 10.1038/s41598-024-79148-x

**Published:** 2024-12-16

**Authors:** Fatma A. Hamada, Saleh S. Sabah, Ehab M.B. Mahdy, Hany S. Abd El-Raouf, Ahmed M. El-Taher, Omneya F.A. El-leel, Ashwaq T. Althobaiti, Mosad A. Ghareeb, Reena Randhir, Timothy O. Randhir

**Affiliations:** 1 Botany Department, Faculty of Science, Aswan University, Aswan, 81528 Egypt; 2 Desertification Department, College of Agriculture, Al-Muthanna University, Samawah, Iraq; 3 National Gene Bank (NGB), Agricultural Research Center (ARC), Giza, 12619 Egypt; 4 Department of Agricultural Botany, Agriculture Faculty, Al-Azhar University, Cairo, 11651 Egypt; 5 Medicinal and Aromatic Plants Dept, Horticultural Research Institute (HRI), Agricultural Research Center (ARC), Giza, Egypt; 6 Department of Biology, College of Science, Taif University, P.O. Box 11099, Taif, 21944 Saudi Arabia; 7 Department of Medicinal Chemistry, Theodor Bilharz Research Institute, Kornaish El Nile, Imbaba, 12411 Giza Egypt; 8 Department of Biology, Springfield Technical Community College, Springfield, MA 01105 USA; 9 Department of Environmental Conservation, University of Massachusetts, Amherst, MA 01003 USA

**Keywords:** *Moringa*, Phytochemistry, Phenological description, Molecular verification, GC-MS analysis, Biochemistry, Molecular biology, Plant sciences

## Abstract

*Moringa* is the sole genus in the family *Moringaceae* used for medicinal and nutrient purposes. Morphological features, phytochemical attributes, and molecular characterization were used for the genetic association and classification among *Moringa oleifera*, *M. peregrina*, and *M. stenopetala*. *Moringa peregrina* recorded a similarity of 84% lonely and placed *M. stenopetala* with *M. oleifera* into a cluster score with a similarity of 95.3%. *M. peregrina* is characterized by phenolic content (243 mg/100 g), flavonoids (7 mg/100 g), and antioxidant activity (1226.85 mg/100 g). GC-MS analysis revealed that *M. oleifera* contained twenty compounds with 2-decenal (E) (39.14%), 2-undecenal (15.51%), nonanal (3.60%), and 2-octenal, (E) (2.48%), while *M. peregrina* identified eighteen compounds with 2-decenal (Z) (25.42%), 2-docecen-1-al (9.35%), and 13-Docosenoic acid, methyl ester, (Z) (4.16%). *M. stenopetala* identified fifteen compounds containing 2-decenal (E) (26.67%), 2-undecenal (24.10%), and nonanal (4.40%). A broad sense of similarity has been scored between *M. oleifera* and *M. stenopetala* by the phytochemical compositions, especially the similarity in the main compounds such as 2-decenal (E), 2-undecenal, and nonanal. It can be concluded that efforts need to be expanded to pay attention to study *Moringa* taxa, due to the rarity of *Moringa peregrina*, and the focus should be on sustainable utilization and conservation. The potential of these taxa would greatly benefit indigenous species in terms of their maintenance, and there is a need for more comprehensive bio-prospecting studies. Therefore, this study evaluates the variability among *Moringa* and highlights the significance of leaf and seed ultrastructure to provide more information and evaluate potential approaches.

## Introduction

*Moringa* is the sole genus in the family Moringaceae. It contains 13 species, which are distributed in Africa, Arabia, Southeast Asia, and South America^1,2^ and cultivated in all tropical and subtropical areas^3–5^. The *M. oleifera* Lam. and *M. peregrina* (Forssk.) Fiori are the most economical species in agricultural and medicinal uses^6,7^. *Moringa* is one of the groups producing mustard oil (glucosinolates), i.e., Brassicaceae and Caricaceae^8^. Generally, plants in the *Moringa* genus are used for various applications and utilizations, including animal feed, human consumption, medicinal use, fertilization, antimicrobial agents, biofuel and biogas production, purification of water, foliar nutrients, green manures, and ornamental purposes^9–15^. *M. oleifera* and *M. peregrina* are widely cultivated and studied species^11^, in contrast, other species have not been studied in detail, and their potential applications and uses have not been given little research and development^16^. Most research in *Moringa* is concentrated mostly on medical applications^17,18^, anatomical identification of plants^19^, and anti-viral activity^20^. Many authors have used the morphological and anatomical characteristics of plants to identify them^21–23^. Plant breeding efforts have been based on taxonomic identification to attribute diagnostic features to particular or varietal parentage^24^. *M. oleifera*, “the drum stick,” is the most well-known and investigated, particularly of the 13 species of genus *Moringa* used in traditional medicine due to the phytochemicals found in its different parts^25^. *M. stenopetala*, “cabbage tree,” is important as it is a major drought-resistant vegetable plant with medicinal and nutritional properties similar to or surpassing those found in *M. oleifera.* However, little research and development has been done on this species^15,26,27^. *M. peregrina* “the Arabian tree of Moringa” is one of the most economically important and valuable medicinal plants in the Egyptian desert. It is threatened by vulnerable habitat, over-grazing as fuel and seed collection, emphasizing the need for habitat protection and conservation^15,28^.

The *Moringa* is known for its quick growth rate, reaching up to 10–12 m in height, and adapts well to humid and dry climates. When ripe, it is identified by its pinnate leaves and long, woody pods because it opens into three valves containing globular seeds. The nutritional and medical benefits of *Moringa*, as well as its usage as a decorative plant and for animal feed, have drawn the attention of farmers, scientists, and researchers^7,29^. *Moringa* has bisexual flowers that are highly pollinated, and pollination is mainly facilitated by animals such as bees and sunbirds. The seeds are also strongly winged, which may allow them to be spread short distances from the parent tree by wind, helping pollination^30^. The *Moringa oleifera* plant grows quickly; it can reach a height of 12 m at the crown and, depending on the cultivar, begins to produce its first fruit 6 to 12 months after planting. In many regions worldwide, trees can flower and bear fruit in one year, and multiple seed harvests are possible. Fruits (pods) ripen about three months after flowering and have to be collected promptly. Each pod contains approximately 26 seeds with a diameter of 1 cm^7,31^.

Natural products extracted from medicinal plants offer the potential for the discovery of new drugs because of their chemical diversity and wide availability. The medical usefulness of these plants results from the bioactive phytochemicals that have specific physiological functions in humans. Alkaloids, flavonoids, tannins, terpenoids, saponins, and phenolic compounds are well-documented as major secondary metabolites^32,33^ which provide a broad spectrum of biological activities like antioxidant, anticancer, antiviral, anti-inflammatory, antiviral, and antimicrobial properties. Contextually, the chemical diversity of these secondary metabolites and their unique chemical structures also had a major impact on biological activities. It is worth noting that the biological activities of plant extracts may be due to a single compound or to the participation of several compounds together in what is known as synergistic activity. In addition, they play an important role in plant’s defense mechanism to mitigate harmful environmental effects. Moreover, by oxidation of fatty acids these phenolic compounds prevent detrimental changes in living organisms^34,35^. *M. oleifera* is one of the traditional medicinal plants, popularly called drumstick tree or horseradish tree, has been widely researched for medicinal uses of bioactive compounds isolated from the plant. All plant parts are used for either human and livestock uses. *Moringa* leaves are consumed as food as well as nutritional supplements. In spite of their beneficial uses, studies on these bioactive compounds of *Moringa* are limited in literature.

Antioxidants are of crucial importance in preserving food by avoiding oxidative damage and deterioration in food quality^36^. Prior studies conducted on *M. peregrina* seeds concentrated on the composition of fatty acid and fixed oil content^37^. However, published studies on the essential oil content of *M. peregrina* seeds are limited. Hence, the current study aims at investigating the chemical constituents and antioxidant activity of oil extracted from *M. peregrina* seeds. This study reports antioxidant properties of *Moringa* seeds using hexane and ethanolic extracts.

Morphological and genetic diversity assessments are important for plant improvement programs. The majority of studies evaluated the genetic diversity of *M. oleifera* using various techniques to assess inter-species variations^38,39^. However, studies on local genotypes of *M. peregrina* are limited. Variations in *M. peregrina* were reported for plants grown in the South Sinai of Egypt^40^ and Western Saudi Arabia^41^.

Multi-locus DNA markers, such as inter-simple sequence repeat (ISSR) and start codon target (SCoT), are simple and common PCR-based techniques and are largely used for determining genetic diversity^42–44^. Start codon targeted polymorphism (SCoT) is a PCR-based technique depending on the short-conserved region of plant genes surrounding the ATG translation start codon^45,46^ using single primers. Hence, this SCoT technique is similar to RAPD or ISSR because a single primer is used in all these techniques^47,48^.

Understanding the genetic diversity of a species in a district is essential when developing management strategies for conservation and improvement activities. Estimation of *Moringa* diversity estimation is significant in clarifying the relationships between individuals within and among the different populations, which impacts conservation management greatly. The general objective of this study is to evaluate the variability among three *Moringa* species: *M. oleifera*,* M. peregrina*, and *M. stenopetala*. The leaves and seeds of *Moringa* sp. were investigated using morphological characterization, SEM (scanning electron microscopy) examination, and numerical analysis of the studied characteristics. This study highlighted the significance of the leaf and seed ultrastructure in exploring species variability. This study highlights the different approaches, viz., micro- and macro-morphological, chemical, and molecular attributes in discrimination and valuation to determine the genetic relationship and classification among species. The attributes generated and provided more information on the association of *Moringa* species. The current study aims to classify the investigated three species of *Moringa*. This study documents morphological and genetic differences among the studied *Moringa* species.

## Materials and methods

### Plant materials

Samples of the studied *Moringa* species were collected from a Moringa collection by Dr. Aboelfetoh M. Abdalla, a botanical garden in Belbes, Al-Sharkia Governorate of Egypt that is a genetic repository for Moringa species in Egypt. Collections were submitted to the procedures and under the authority of the National Gene Bank (NGB), Agricultural Research Center (ARC), Giza, Egypt. The NGB Herbarium conducted the formal identification and referral of plant materials as presented in Table 1. All taxa are deposited in the National Gene Bank (NGB), Agricultural Research Center (ARC), 9 Gamaa St., 12619, Giza, Egypt. All samples were taxonomically checked to validate the identification methods suggested by Bailey^49^. An additional identification check was conducted using the plant materials from the Cairo University Herbarium and The Agriculture Museum in Giza, Egypt. All samples were submitted to the Herbarium at Al-Azhar University on November 17, 2022 and the Herbarium of NGB (Table 1). This study compiles information on species, their locations, the number of individuals collected, their distribution, conservation status, and evaluation.

Thirty-one morphological traits of the leaf and seed were scored and coded to build a numerical data matrix. Statistical analysis with PRIMER (Software, Version 6.0; https://www.primer-e.com*)* was used to compare the investigated taxa. Five individuals were used to represent each species in estimating the mean and standard error (SE) for quantitative traits and used in the numerical analysis of traits. All the experiments were performed following the relevant guidelines and legislations of the National Gene Bank (NGB).

**Table 1:** Studied *Moringa* species collected from Al-Sharkia Governorate and their voucher specimens at Al-Azhar University by Dr. A. El-Taher, Al-Azhar University, Cairo, Egypt.

ID	Species	AN	Evaluation	Distribution	Conservation		
					Botanic Gardens	Herbaria	Gene Bank
1	*Moringa peregrina (*Forssk.) Fiori	M0065W	VU	Da, R, GE, S	-	ALAU, HNGB	EDGB NGB
2	*Moringa oleifera* Lam.	M0066C	VC	BG	OR, NGB, ShM	ALAU, HNGB	NGB
3	*Moringa stenopetala* (Baker f.) Cufod.	M0067C	C	BG	ShM	ALAU, HNGB	-

AN = accession number deposited in NGB Herbarium. Five trees representing each species were collected. Distributions: Da = Arabian desert, east of Nile, R = Red Sea, GE = Gebel Elba, S = Saini proper, BG = Botanical Gardens; Evaluation: VU = Vulnerable, C = Common, VC = Very common; Botanic Gardens: OR = Orman botanic Garden, ShM = Shehab Mazhar botanic Garden, HNGB = Herbarium of NGB, Agricultural Research Center (ARC), ALAU = Botany Department, Faculty of Agriculture, Al-Azhar University, Gene banks: NGB = National Gene Bank, Agricultural Research Center, EDGB = Egyptian Desert Gene Bank, North Sinai.

### Phytochemical analysis

#### Phytochemical constituents

The protocol devised by Nagata and Yamashita^50^ was followed to determine chlorophyll a, b, and carotenoid contents. The leaf samples (0.2 g) of *Moringa* were ground in acetone (80%) and then filtered using Whatman glass microfiber filters (GF/F filter discs: 0.7 μm pore size).

The absorbance of the extract was recorded at wave lengths of 663, 645, and 446 nm by using a UV-spectrophotometer.

Chlorophyll a and b, as well as carotenoid contents, were calculated using the following equations (I-III):

I. Chlorophyll a = 0.999 × A663–0.0989 × A645.
II. Chlorophyll b = -0.328 × A663 + 1.77 × A645.
III. Carotenoid = (0.216 × A663–1.22 × A645) – (0.304 × A505 + 0.452 × A453).

Where, A663, A645, A505, and A453 are the readings at wavelengths of 663, 645, 505, and 446 nm, respectively^50^.

Two grams of green leaves were finely ground, after that the dry powder was defatted using petroleum ether (60–80 °C), subsequently the residue was extracted with 20 ml methanol (80%). Then, the extract was filtered. All measurements were conducted using an ultraviolet-visible spectrophotometer-MA9523-SPEKOL 211 (ISKRA, Slovenia). The total phenolic content (TPC) of the extract was determined following Singleton et al.^51^. Woisky and Salation^52^ method was used to estimate the total flavonoid content (TFC). The total antioxidant capacity (TAC) of the extracts was determined according to Prieto et al.^53^, whereas ascorbic acid was estimated using the procedure of Klein and Perry^54^. The absorbances were measured at 765 nm, 415 nm, and 695 nm for TPC, TFC, and TAC, respectively.

####  GC-MS analysis

#####  Extraction

Ten grams (10 g) from each seed dry powder were extracted separately with 150 ml *n*-hexane at room temperature with shaking daily, followed by filtration and extraction again four times. Then, each extract was filtered using Whatman filter paper No.1 and concentrated by using a rotatory evaporator (Buchi, Switzerland) at (40ºC). The obtained extracts were collected and stored at room temperature in the dark for further processing.

##### Analysis

Gas chromatography-mass spectrometry (GC-MS) analysis was conducted using a Thermo Scientific, Trace GC Ultra/ISQ Single Quadrupole MS instrument coupled with TG-5MS fused silica capillary column (30 m, 0.251 mm, 0.1 mm film thickness). A 70 eV electron ionization system was used, with Helium gas serving as the carrier gas at a flow rate of 1 ml/min for the GC-MS analysis. The temperature for both MS transfer line and injector was adjusted to 280 °C. The oven temperature was set to 50 °C initially, with a hold time of 2 min. It then increased to 150 °C at a rate of 7 °C per minute, then to 270 °C at a rate of 5 °C per minute, with a hold time of 2 min. Finally, it reached 310 °C as the final temperature, with a hold time of 10 min, and increased at a rate of 3.5 °C per minute. The investigation focused on quantifying all the detected components by calculating the percentage relative peak area. The compounds were tentatively identified by comparing their retention time and mass spectra with the NIST and WIL-LY library data acquired from the GC-MS instrument^55–58^.

### Molecular analysis

####  Start codon target (SCoT) markers

Ten fresh leaflets for each taxon were collected in silica gel bags (5:1 silica gel: tissue) for the step of DNA extraction. The samples were powdered using immersion in liquid nitrogen and then crushed via a sterile mortar and pestle. The isolation was done using the DNeasy plant mini kit (Biobasic) and stored at − 80 °C for subsequent steps. The quality was checked using a Nanodrop 8000 Spectrophotometer (Thermo Scientific, Wilmington, USA). The DNA extraction was done according to Mahdy et al.^59^.

Fifteen SCoT primers (Table 2) were used according to the design of Collard and Mackill^60^ and procured from Biobasic Com. PCR amplification was performed using Techni TC-512 Thermal Cycler. The temperature and time were programmed as one cycle at 94 °C for 4 min, then 40 cycles for one minute at 94 °C, 1 min for annealing at 57 °C and two minutes at 72 °C, and finally, 72 °C for 10 min. The PCR products were stored at 4 °C for further analysis. The products were screened on a 1.2% agarose gel staining with ethidium bromide. A ladder marker of 100 bp was used. The run was conducted for 30 min at 100 V in mini-submarine gel BioRad. Gels were photographed by a transilluminator and analyzed by Bio-Rad video Gel documentation 2000. Profiles were recorded as 1 if present or 0 if absent on standard marker via Alpha Ease FCTM (version 4.9.1) software.

**Table 2:** SCoT primers used and their nucleotide sequences.

Primer	Sequence	Primer	Sequence
SCoT2	ACCATGGCTACCACCGGC	SCoT11	ACAATGGCTACCACTACC
SCoT3	ACGACATGGCGACCCACA	SCoT12	CAACAATGGCTACCACCG
SCoT4	ACCATGGCTACCACCGCA	SCoT13	ACCATGGCTACCACGGCA
SCoT5	CAATGGCTACCACTAGCG	SCoT14	ACCATGGCTACCAGCGCG
SCoT6	CAATGGCTACCACTACAG	SCoT15	CCATGGCTACCACCGGCT
SCoT7	ACAATGGCTACCACTGAC	SCoT16	CCATGGCTACCACCGGCA
SCoT9	ACAATGGCTACCACTGCC	SCoT18	CCATGGCTACCACTAGCA
SCoT10	ACAATGGCTACCACCAGC		

Banding profiles were recorded as one if present or zero if absent based on standard marker using Alpha Ease FCTM (version 4.0.1) software. Genetic similarity was determined using the Jaccard coefficient^61^. Algorithms of the un-weighted pair group method with arithmetic (UPGMA) averages are used to build trees^62^ to study the relationship among populations.

### Statistical analysis

A randomized complete block design (RCBD) was used for the experimental design. Data were analyzed using the factorial method of Sendecor and Cochran^63^ where the means were compared using the Least Significant Difference (LSD). The DNA bands produced by each primer were counted, and the molecular sizes of these bands were compared with those of the ladder DNA markers. The bands scored from DNA banding patterns generated by the primers were pooled together. Then, the presence or absence of each DNA band was recorded as a binary character in a data matrix (coded 1 (present) and 0 (absent) to calculate genetic similarity. Calculations were done using Dice similarity coefficients^64^ by using the SPSS software (v. 10). The genetic similarity matrix was then used to construct a dendrogram tree among the studied *Moringa* species using PRIMER software (www.primer-e.com*).*

## Results and discussion

Morphological traits of leaves, seeds, chemical features, and molecular markers were examined to assess the taxonomic relationship between the three species of the genus *Moringa* under study: *M. peregrina*, *M. oleifera*, and *M. stenopetala*. The numerical analysis method utilizing these attributes was also applied to assess how similar or unlike these species are to one another.

###  Leaf and seed morphology

**Fig. 1:**
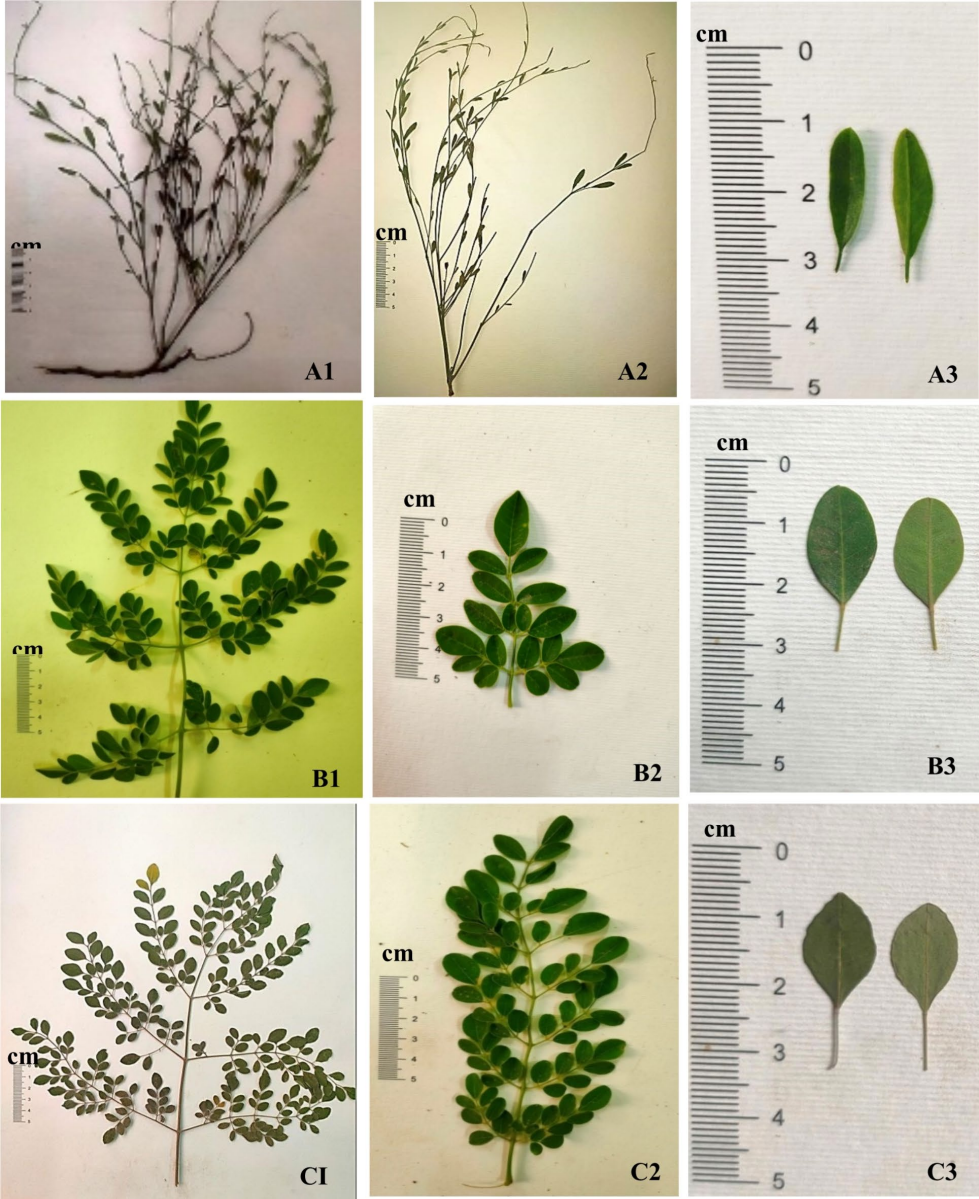
Leaf and leaflet morphology of *Moringa* species: (**A**) *Moringa peregrina*, (**B**) *Moringa oleifera* and (**C**) *Moringa stenopetala*. Photographs were taken by the co-author, Prof. Dr. Ahmed M. El-Taher (*eltaher69@azhar.edu.eg*).

**Fig. 2:**
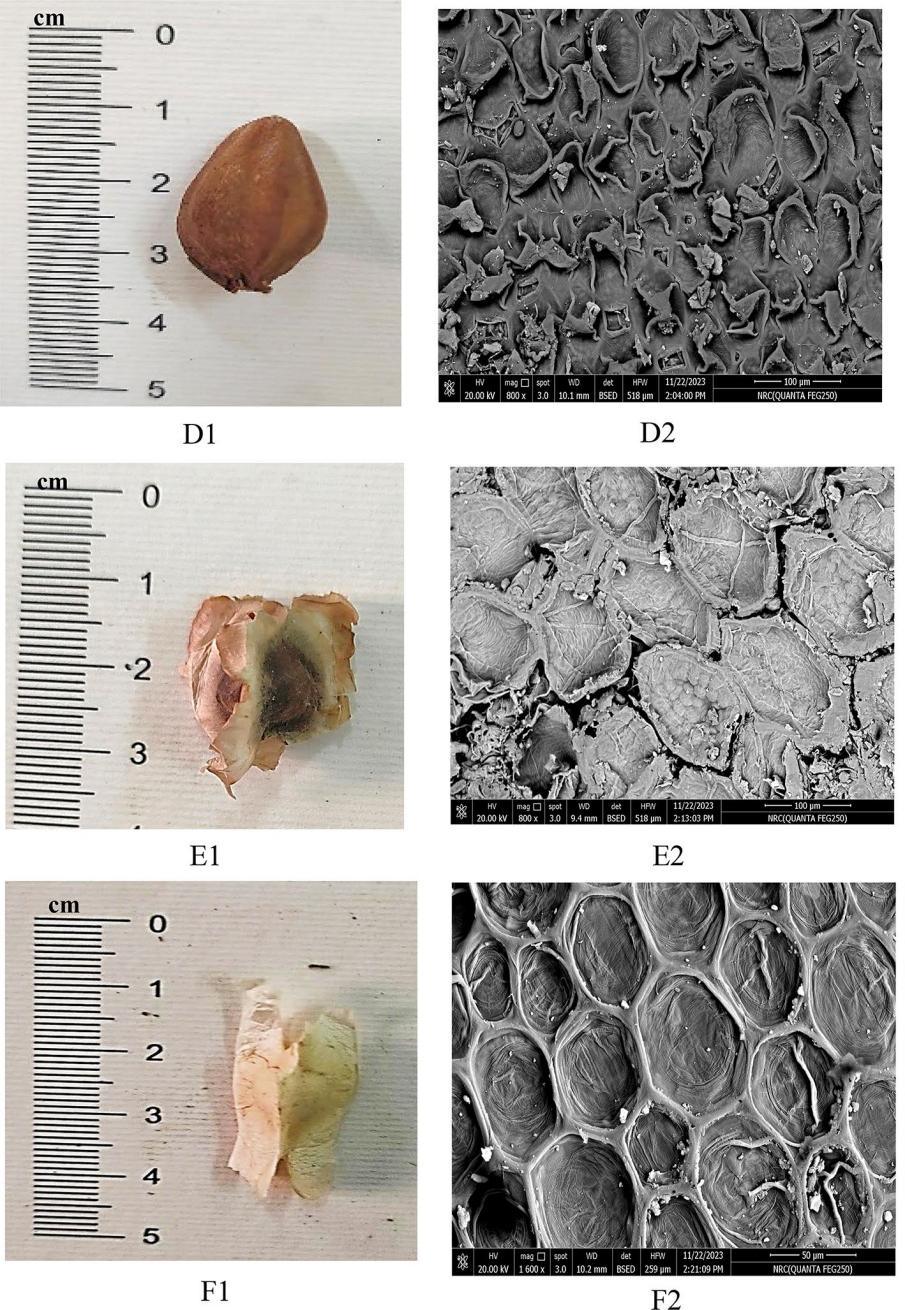
Microphotographs of seed morphology of the *Moringa* studied species: D1-D2. *Moringa peregrina*, E1-E2. *Moringa oleifera* and F1-F2. *Moringa stenopetala*. Photographs were taken by the co-author, Prof. Dr. Ahmed M. El-Taher (*eltaher69@azhar.edu.eg*).

**Fig. 3:**
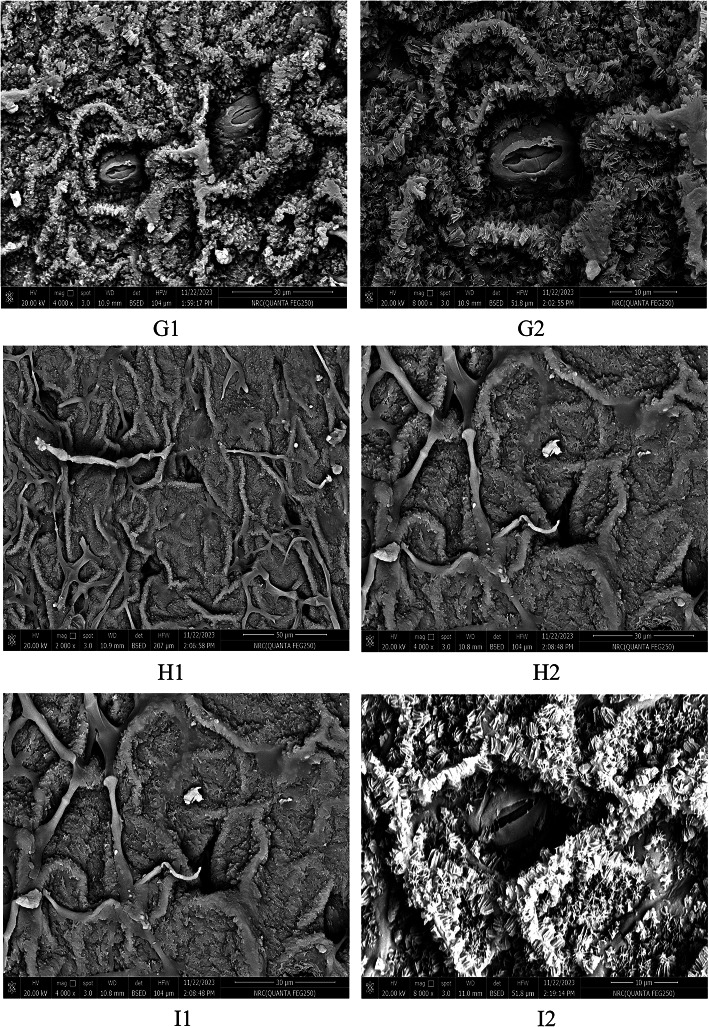
Microphotographs of leaf epidermal of the *Moringa* studied species: G1-G2. *Moringa peregrina*, H1-H2. *Moringa oleifera* and I1-I2. *Moringa stenopetala*.

The growth forms perennial shrubs; the stem is erect, much branched, and cylindrical in the studied species. The leaflet in the studied species was examined concerning shape, type, texture, apex, and the number of leaflets (Table 3; Fig. 1). The leaflet is sub sessile in the studied species; the petiole surface is sparsely hairy in *M. peregrina* and pubescent in *M. oleifera* and *M. stenopetala*. The leaves of the studied species are compound and recorded in two types: pinnate in *M. peregrina* and imparipinnate in *M. oleifera* and *M. stenopetala*. The leaflets of all species are entire, and for blade outline (leaflet shape), two main types were recorded: oblong, found in *M. peregrina*, and elliptic-obovate, found in *M. oleifera* and *M. stenopetala.* Leaflet apices ranged from acute in *Moringa peregrina*, to emarginate in *M. oleifera*. We also observed an obtuse-emarginated apex in *M. stenopetala.* Leaflets are glabrous in the upper leaf in the studied species. In contrast, they are in the lower leaf sparsely hairy in *M. peregrina* and pubescent in *M. oleifera* and *M. stenopetala*. The leaflet length is (1.7–2.1 cm) in *M. peregrina*, (1.5–2.3 cm) in *M. oleifera* and *M. stenopetala*. The leaflet width is (0.4–0.5 cm) in *M. peregrina*, and (1–1.5 cm) in *M. oleifera* and *M. stenopetala*. Seed shape (Fig. 2) is ovoid-trigonous in *M. peregrina*, globose or sub-globose with three papery wings in *M. oleifera*, and elliptical in *M. stenopetala*. Seed color is brown in *M. peregrina* and *M. oleifera* and creamy white in *M. stenopetala*. Seed surface sculpture is reticulate and anticlinal wall shape straight in studied species. Seed length varied among the studied species; 1.7–2.1 cm in *M. peregrine*, 2.5–2.7 cm in *M. oleifera*, and 2.3–2.7 cm in *M. stenopetala*. Seed width varied among the studied species; 1.2–1.5 cm in *M. peregrina*, 2–2.6 cm in *M. oleifera*, and 1.2–1.5 cm in *M. stenopetala*. The anticlinal wall shape of the seed is straight or undulated in *M. peregrina* and straight in *M. oleifera* and *Moringa stenopetala*, anticlinal walls raised in *M. peregrine*, *M. oleifera*, and *M. stenopetala*, outer periclinal walls convex in *M. peregrina* concave in *M. oleifera* and *M. stenopetala*, inner periclinal walls concave in *M. peregrine*, *M. oleifera*, and *M. stenopetala*. Epidermal cell wall straight to undulate, stomata leveling depressed and stomata type anomocytic in *M. peregrine*, *M. oleifera*, and *M. stenopetala* (Table 3; Fig. 3).

Our results indicated that the growth forms perennial shrubs; the stem is erect, much-branched, and cylindrical in the studied species. The leaflet in the studied species was examined in terms of shape, type, texture, apex, and number of leaflets. The leaflet is subsessile in the studied species; the petiole surface is sparsely hairy in *M. peregrina* and pubescent in *M. oleifera* and *M. stenopetala*. The leaves of the studied species are compound and recorded in two types: pinnate in *M. peregrina* and imparipinnate in *M. oleifera* and *M. stenopetala*. The leaflets of all species are entire and for blade outline (leaflet shape), oblong, in *M. peregrina* and elliptic-obovate in *M. oleifera* and *M. stenopetala.* Leaflet apices ranged from acute in *M. peregrina*, to emarginate in *M. oleifera*, and obtuse-emarginated apex in *M. stenopetala.* Leaflets are glabrous in the upper leaf whereas in the lower leaf sparsely hairy in *M. peregrina* and pubescent in *M. oleifera* and *M. stenopetala*. These were found to be important diagnostic characteristics as they conformed with the results obtained by Azza^65^, who reported that *M. oleifera* leaflet shape is obocordate, with emarginate apex, symmetric base, leaflet upper surface hairy, colliculate sculpture of leaflet upper surface, tuberculate-reticulate on lower one, also Rangnath et al.^66^ reported that *M. oleifera* leaves generally tripinnate, pinnate and pinnules opposite, evanescent; circulars − 2 cm long and 0.6–1 cm wide. The side elliptic, the terminal obviates. *M. stenopetala* leaves imparipinnate. The shape is the elliptic, obtuse apex, symmetric base. The leaflet’s upper surface is a hairy, lower smooth, rugose sculpture of the leaflet surface upper, reticulate-verrucate on the lower one. *M. peregrina* leaves are pinnate, leaflet shape is linear, acute apex, symmetric base, hairy leaflet upper surface (non-glandular, glandular), smooth lower surface, anomocytic stomata on the upper epidermis at superficial level, unclear on lower one, the rugose-tuberculate sculpture of upper leaflet surface, and rugose-tuberculate on lower one. Also, Zhigila^67^ observed that the leaves in *M. oleifera* varieties are alternate, composite, bipinnate, or tripinnate, with 2 to 6 pairs of opposite pinnae bearing opposite leaflets in 3 to 7 pairs. It also has a broader terminal leaflet, green leaf lamina color, and an entire leaflet margin.

The seed shape is ovoid-trigonous in *M. peregrina*, globose or sub-globose with three papery wings in *M. oleifera*, and elliptical in *M. stenopetala*. The seed color is brown in *M. peregrina* and *M. oleifera* and creamy white in *M. stenopetala*. Seed surface sculpture is reticulate and anticlinal wall shape straight in the studied species. The anticlinal wall shape of the seed is straight or undulated in *M. peregrina* and straight in *M. oleifera* and *M. stenopetala*, anticlinal walls raised in *M. peregrine*, *M. oleifera*, and *M. stenopetala*, outer periclinal walls convex in *M. peregrina* concave in *M. oleifera* and the *M. stenopetala*, inner periclinal walls concave in *M. peregrine*, *M. oleifera* and *M. stenopetala*. Epidermal cell wall straight to undulate, stomata leveling depressed and stomata type anomocytic in *M. peregrine*, *M. oleifera*, and *M. stenopetala*. These results are in agreement with Azza^65^ who characterized *M. oleifera* as seeds are brown, round with tan edges, rough texture, reticulate epidermal cell walls, anticlinal walls raised (4–6 gonal)-straight, and concave outer periclinal walls. *M. stenopetala* seeds are brown with rough texture, reticulate-foveate epidermal cell walls, raised anticlinal walls (5–6 gonal)-straight, and concave outer periclinal walls.

**Table 3:** Morphological descriptions of leaves and seeds of the three Morigna species.

Characters		Species		
		M. peregrina	M. oleifera	M. stenopetala
Duration		Perennial	Perennial	Perennial
Habit		Shrub	Shrub	Shrub
Stem Branching		Much branched	Much branched	Much branched
Outline of internodes		Cylindrical	Cylindrical	Cylindrical
Leaf morphology	Leaf type	Pinnate	Imparipinnate	Imparipinnate
	Leaflet arrangement	Opposite	Opposite	Opposite
	Upper leaflet texture	Smooth	Smooth	Smooth
	Lower leaflet texture	Hairy	Hairy	Hairy
	Leaflet shape	Oblong	Elliptic-Obovate	Elliptic- Obovate
	Leaflet apex	Acute	Emarginate	Obtuse- Emarginate
	Leaflet margin	Entire	Entire	Entire
	leaflet base	±Symmetric	±Symmetric	±Symmetric
	Leaflet length (cm) Mean ± SD.	1.7–2.1 1.9 ± 0.16	1.5–2.3 2 ± 0.30	1.5-2 1.75 ± 0.18
	Leaflet width (cm) Mean ± SD.	0.3–0.5 0.4 ± 0.1	1-1.4 1.2 ± 0.16	1-1.5 1.24 ± 0.18
	Leaflet number/leaf	6	9–11	5–7
	leaflet petiole	Sub sessile	Sub sessile	Sub sessile
	Petiole surface	Sparsely hairy	Pubescent	Pubescent
	Petiole out line	Terete	Terete	Terete
	Petiole length cm Mean ± SD.	0.2–0.5 0.35 ± 0.11	0.2–0.7 0.42 ± 0.17	0.2–0.7 0.42 ± 0.17
	Rachis outline	Terete	Terete	Terete
	Rachis surface	Hairy	Hairy	Hairy
Seed morphology	Seed shape	Ovoid trigonous	Globose or sub-globose	Elliptical
	Seed wing	Absent	Present	Present
	Wing form	Not applicable	Papery	Papery
	Seed color	Brown	Brown	White Creamy
	Seed length (cm)	1.7–2.1	2.5–2.7	2.3–2.7
	Seed width (cm)	1.2–1.5	2-2.6	1.2–1.5
	Seed texture	Glabrous	Glabrous	Glabrous
	Surface sculpture	Colliculate	Reticulate	Reticulate
	Anticlinal wall shape	Straight or undulate	Straight	Straight
	Anticlinal walls	Raised	Raised	Raised
	Outer periclinal walls	Convex	Concave	Concave
	Inner periclinal walls	Concave	Concave	Concave
Epidermal	Epidermal cell wall	Straight to undulate	Straight to undulate	Straight to undulate
	Stomata leveling	depressed level	depressed level	depressed level
	Stomata type	Anomocytic	Anomocytic	Anomocytic

20

###  Numerical analysis

**Fig. 4:**
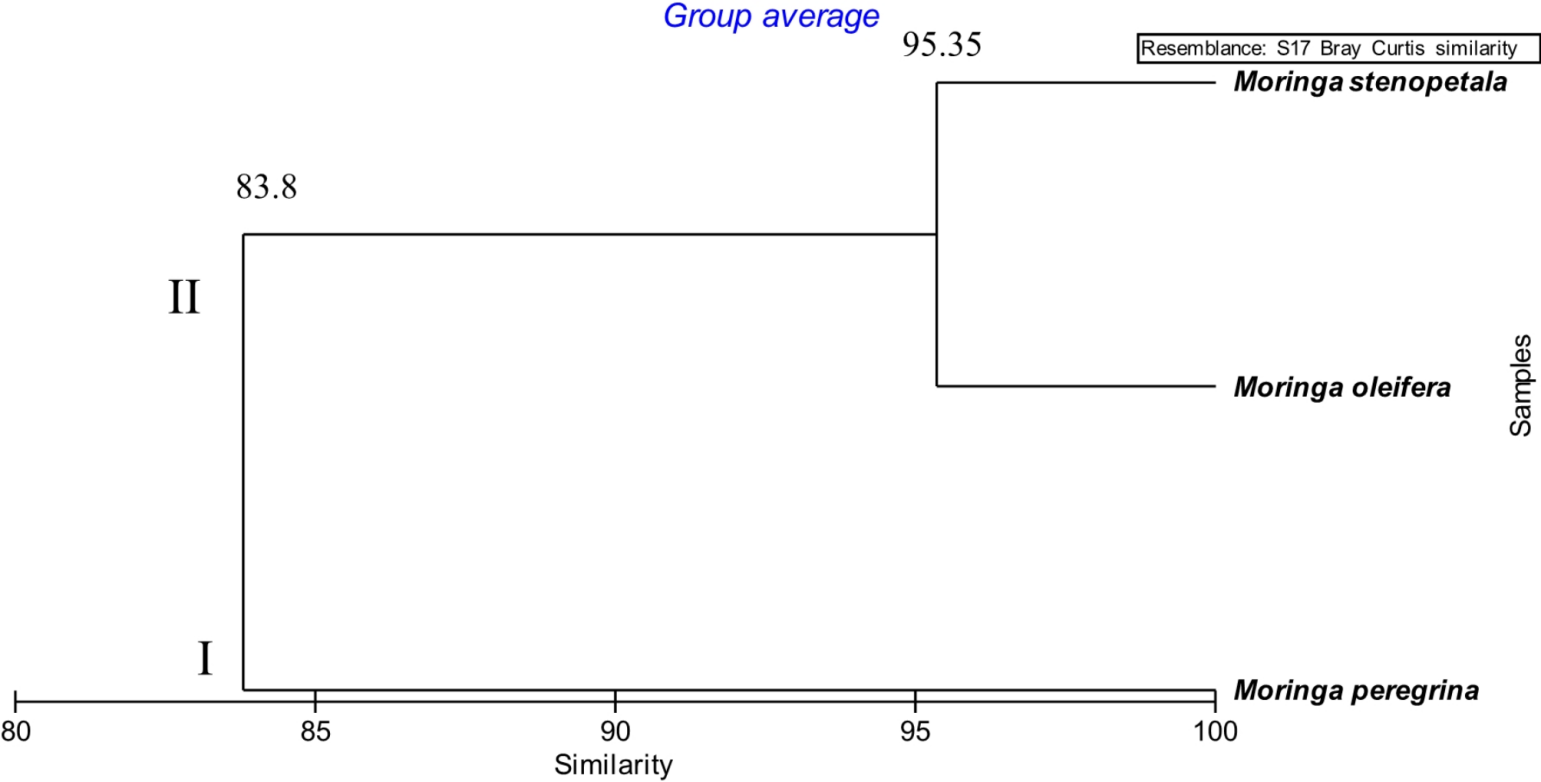
Dendrogram showing the interrelationships between three species of *Moringa* based on 31 morphological characters by using the Primer program (www.primer-e.com).

Taxonomic relatedness of the studied species was analyzed using morphological characterization. The numerical analysis was conducted using the attributes of *Moringa* species, including their 31 morphological characteristics. A dendrogram was constructed and revealed two main clusters. *M. peregrina* was separated in the first cluster at an 83.8% similarity level. The second cluster comprised *M. oleifera* and *M. stenopetala* at a 95.35% similarity level (Fig. 4).

The *M. oleifera* and *M. stenopetala* are more related and separated in cluster I, and *M. peregrina* is separated in cluster II. This study agrees with the results of Azza^65^ who observed that the studied species can be split into two main clusters based on similarity or dissimilarity distance. The first cluster included both *M. oleifera* and *M. stenopetala*, linked together at a 0.5 similarity level. Whereas, the second cluster included a single species, *M. peregrina*, (with a similarity level of 2.0). All three species are linked in the main cluster at similarity level of 2.0. This is attributed to their belonging to the *Moringa* genus.

### Phytochemical study

#### Chemical constituents

Methanolic extracts of leaves were derived to determine and discriminate the presence of significant secondary metabolites in three *Moringa* species under study. Table 4 presents the phytochemical contents (mg/100 g) for three *Moringa* species. The highest content of chlorophyll a and b were found in M. *oleifera* (40.98 and 12.2 mg/100 g), followed by *stenopetala* (40.19 and 11.01 mg/100 g) and *M. peregrina* (29.2 and 3.57 mg/100 g and), respectively. While, the *M. peregrina* contains the highest content of phenols (243 mg/100 g) and antioxidant capacity (1226.85 mg/100 g), *M. peregrina* is characterized by the highest flavonoid content (7 mg/100 g) only.

**Table 4:** Chemical contents (mg/100 g) for leaves of *Moringa* species under study.

Species	Chl. a	Chl. B	Carotenoids	Total Phenols	Total Flavonoids	Total Antioxidant Capacity	Vit. C
M. oleifera	40.98	12.20	71.28	241.05	6.06	745.64	120.87
M. stenopetala	40.19	11.01	69.83	243.00	3.05	1226.75	110.85
M. peregrina	29.20	3.57	44.28	200.12	7.00	1066.39	111.72
LSD (99%)	0.13	0.18	3.87	0.56	1.90	6.68	6.12
LSD (95%)	0.08	0.12	2.55	0.37	1.25	4.41	4.04

#### GC-MS analysis of *Moringa* species seed extracts

**Fig. 5:**
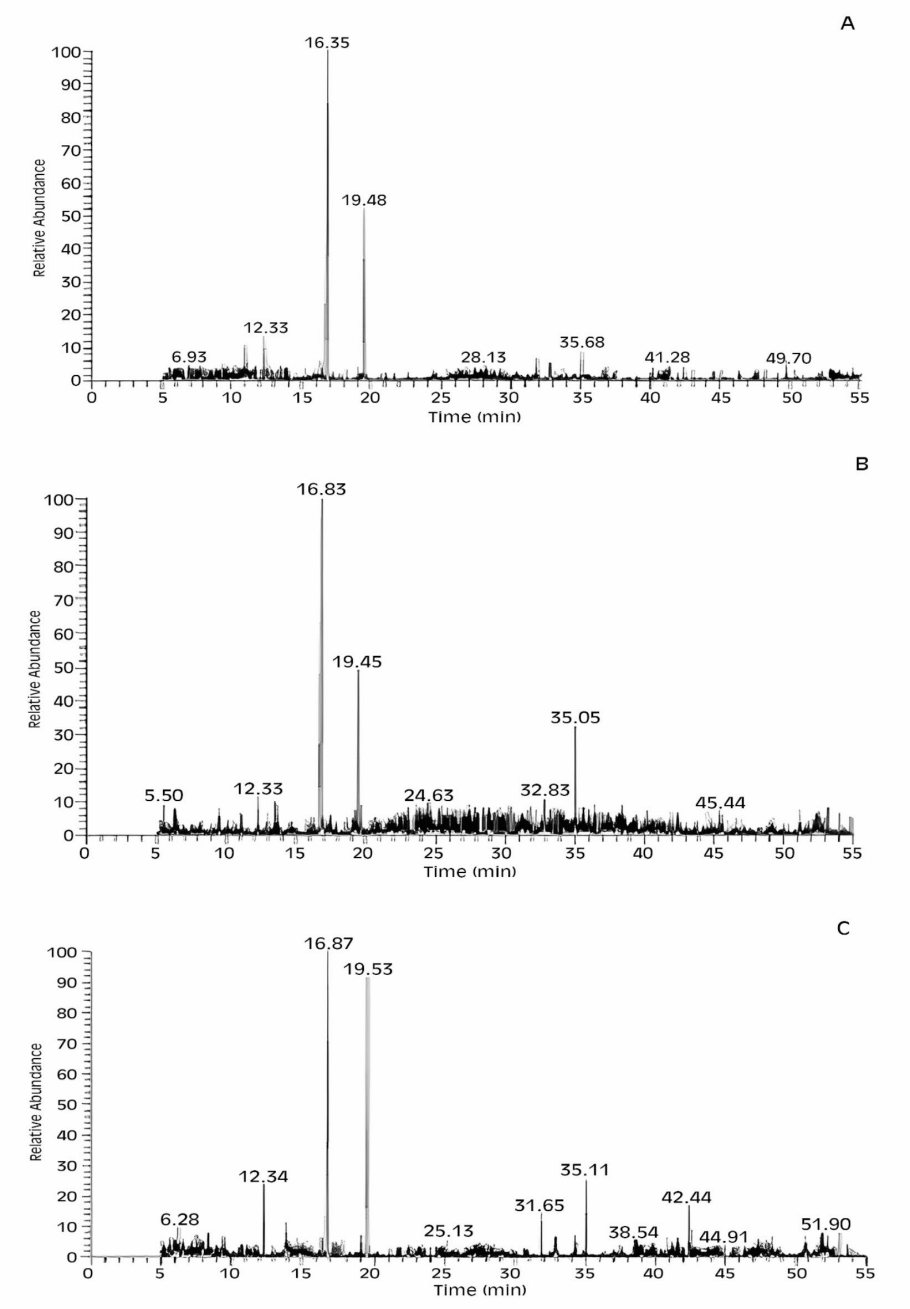
GC-MS chromatograms of the investigated *Moringa* species seed extracts; 1 A) *M. oleifera*, 1 B) *M. peregrina*, and 1 C) *M. stenopetala*.

GC-MS analysis of *M. oleifera* seed *n*-hexane extract identified twenty compounds (Table 5; Fig. 5A) representing 75.27% of the total extract composition. The main identified compounds are 2-decenal, (E) (39.14%), 2-undecenal (15.51%), nonanal (3.60%), and 2-octenal, (E) (2.48%). While, GC-MS analysis of *M. peregrina* seed *n*-hexane extract led to the identification of eighteen compounds (Table 6; Fig. 5B), representing 55.89% of the total extract composition. The main identified compounds are 2-decenal, (Z) (25.42%), 2-docecen-1-al (9.35%), and 13-Docosenoic acid, methyl ester, (Z) (4.16%). Moreover, GC-MS analysis of *M. stenopetala* seed *n*-hexane extract led to identifying fifteen compounds (Table 7; Fig. 5C) representing 74.95% of the total extract composition. The main identified compounds are 2-decenal, (E) (26.67%), 2-undecenal (24.10%), and nonanal (4.40%). Taken together, the current finding showed that there is a slight variation in the chemical composition between the three investigated *Moringa* species, with a very large similarity between *M. oleifera* and *M. stenopetala* in the chemical composition, especially the similarity in the major compounds [e.g., 2-decenal, (E), 2-undecenal, and nonanal (4.40%)].

**Table 5:** Chemical constituent of *M. Oleifera* seed extract.

No.	*R*_t (min)_	Area %	Identified compound
1	6.12	0.85	N-Acetylaspartylglutamic acid
2	6.40	0.61	1,2-Bis(2-methoxy-1-naphthyl)tetraphenylbenzene isomer
3	7.34	0.96	8,8’-bis(8”-Acetyl-8”,9”-dihydrocaffeine)
4	11.01	2.48	2-Octenal, (E)
5	11.51	1.35	2,3,7,8,12,13,17,18-octapropyl-21 H,23 H-porpin
6	12.33	3.60	Nonanal
7	12.45	0.78	9-Octadecenoic acid (Z)-, 3-[(1-oxohexadecyl)oxy]-2-[(1-oxooctadecyl)oxy]propyl ester
8	13.93	0.60	cis-3-Nonen-1-ol
9	16.85	39.14	2-Decenal, (E)
10	17.71	0.62	2-(3’-Methoxyprop-2’-en-1’-yl)-2-methylcyclopentane-1,3-dione
11	19.47	15.51	2-Undecenal
12	24.49	0.64	(3 S)-3-Acetoxy-á,á-caroten-4-one
13	27.99	0.69	1-(4-Anisyl)-2,5-di(4-(1,3-dioxolan-2-yl)phenyl)-3,4-diphe nyl-1,3-cyclopentadiene
14	31.84	1.37	Dodecanoic acid, methyl ester
15	32.80	1.58	Docosanoic acid
16	35.07	1.51	13-Docosenoic acid, methyl ester, (Z)
17	40.83	0.88	11,23-Di-tertbutyl-5,17-diethoxycarbonyl-25,26,27,28-tetra hydroxycalix[4]arene
18	42.42	0.75	1,2-Benzenedicarboxylic acid, dioctyl ester
19	53.06	0.75	(-)^®^3-azido-5-methyl-2(5 H)-furanone
20	53.28	0.60	6-Pentanoylcyclohex-3-ene-1-carboxylic acid
75.27%			

**Table 6:** Chemical constituent of *M. Peregrina* seed extract.

No.	*R*_t (min)_	Area %	Identified compound
1	6.42	1.24	3,5-Diphenyl-3,5-(9,10-phenanthylene)tricyclo[5.2.1.0]deca ne-4-one-8-exo-9-endodicarboxylic acid
2	11.14	1.02	2,6-Bis(2,3,5-triphenyl-4-oxocyclopentadienyl)pyridine
3	12.33	1.40	Nonanal
4	16.82	25.42	2-Decenal, (Z)
5	19.27	1.01	2-(2-Carboxyvinyl)-5,10,15,20-tetraphenylporphyrin
6	19.45	9.35	2-Docecen-1-al
7	23.64	1.03	N-Acetylaspartylglutamic acid
8	24.53	1.32	(P)-N, N’-Dimethyl[1 + 1]cycloamide
9	27.82	0.98	Rubixanthin acetate
10	28.83	1.13	3(1),5(1)-cyclo-5(1)-(N-methylamino)-2,7,8,12,13,17,18-heptapropyl-3(1)-ethyl-21 H,23 H-porphrin
11	28.91	0.95	Bicyclo[3.3.1]non-6-en-3-ol, 7-methyl-,endo
12	28.98	1.06	Cyclohexane,1,1’,1’’,1’’’-(1,6-hexanediylidene)tetrakis
13	31.84	1.26	Dodecanoic acid, methyl ester
14	34.23	1.55	3-[9,9-Dihexyl-7-(pyridine-2-yl)Fluororen-2-yl]-9-hexyl Carbazole
15	35.08	4.16	13-Docosenoic acid, methyl ester, (Z)
16	36.03	0.93	9-Hexadecenoic acid
17	37.19	1.12	Coumarin
18	42.43	0.96	1,2-Benzenedicarboxylic acid, bis(2-ethylhexyl)ester
55.89%			

**Table 7:** Chemical constituent of *M. Stenopetala* seed extract.

No.	*R*_t (min)_	Area %	Identified compound
1	7.98	1.26	2,11-Dihydroxy3,6,7,10-tetrapentyloxytriphenylene
2	10.74	0.65	Cyclopenta[f]thieno[2,3-b]pyridine
3	11.13	0.66	1-Pentanol, 2-methyl
4	12.33	4.40	Nonanal
5	13.93	2.60	2-(1-Methylethyl)-1,3-propanediol
6	14.05	1.03	(2R)-8,13-epoxy-2,2-(8’,13’-epoxy-2’b-methoxy-3’-oxolabd ane-1’a,2’a-diyldioxy)-1a-hydroxylabdan-3-one
7	16.40	0.95	(2R,4R)-2,4-dimethylhexan-1-ol
8	16.86	26.67	2-Decenal, (E)
9	17.72	1.30	1-Butene, 3,3-dimethyl
10	19.08	0.92	Oxalic acid, cyclohexyl tetradecyl ester
11	19.53	24.10	2-Undecenal
12	31.85	2.16	Dodecanoic acid, methyl ester
13	32.89	1.43	3-Pentanol, 2,4-dimethyl
14	35.11	3.72	9-Octadecenoic acid, methyl ester, (E)
15	42.44	3.10	1,2-Benzenedicarboxylic acid, bis(2-ethylhexyl) ester
74.95%			

The significant difference between leaf and seed extracts is mainly due to the extraction method. The type of solvent used in extraction plays a major role in determining the type of chemical compounds to be extracted. In the present study, the leaves were extracted using polar solvents like acetone and methanol, which resulted in polar extracts comprising some specific chemical classes such as phenols (flavonoids and phenolic acids), anthraquinones, coumarins and other related compounds. On the other hand, extracting seeds using *n*-hexane as a non-polar solvent resulted in targeting a specific class of non-polar compounds like fatty acids and their derivatives. Therefore, we can conclude that the choice of solvent used in extraction has a major impact on the chemical composition and biological activities of the investigated species.

The current study showed that the variation in these phytoconstituents (Tables 5, 6 and 7) in *Moringa* species contributes to the various medicinal uses and biological activities^68^. This is also useful in taxonomical studies for classification and systematics. *Moringa* possesses high antioxidant activity primarily because of its high flavonoid contents in flavanol and glycoside forms^69^. The flavonoids are one of the secondary metabolites that have various bioactivities at harmless concentrations^70^. Dietary flavonoids are valuable in preventing against several cancer diseases^71,72^ and have curative properties against various pathogens. Furthermore, they are traditionally utilized for their analgesic, antimicrobial, and soothing properties^73–75^.

The high antioxidant activity in *Moringa* plants is attributed to their high content of phenolics. For example, a high content occurs in *M. stenopetala* with phenolic content level of 243 mg/100 and antioxidant level of 1226.75 mg/100 g (Table 4). The free radicals produced in cells are stabilized by phenolic compounds by donating or accepting electrons, acting as antioxidants. High concentrations of phenols in the extracts might induce caspase and apoptosis. It corresponds to those of Abdel Baky and El-Baroty^76^. El-Alfy et al.^77^, Teixera et al.^78^, and Saini et al.^79,80^ reported that the major carotenoid content detected in the *M. oleifera* and *M. peregrina*. *Moringa* leaves in general are reported to have relatively high levels of protein, vitamin A, potassium, vitamin C, and calcium^2,81^. Zaghloul et al.^82^ reported that *M. peregrina* was also used as fodder to increase animal weight. *M. oleifera* contains a high amount of zeatin that has been used as a natural plant growth enhancer and helps to increase crop yields^7^. GC-MS technique has been widely used to identify the chemical constituents of *Moringa* species seed extracts. Previous reports revealed the presence of a broad array of volatile and non-volatile compounds among the tested *Moringa* extracts^83–85^.

### Molecular characterization and genetic diversity

**Fig. 6:**
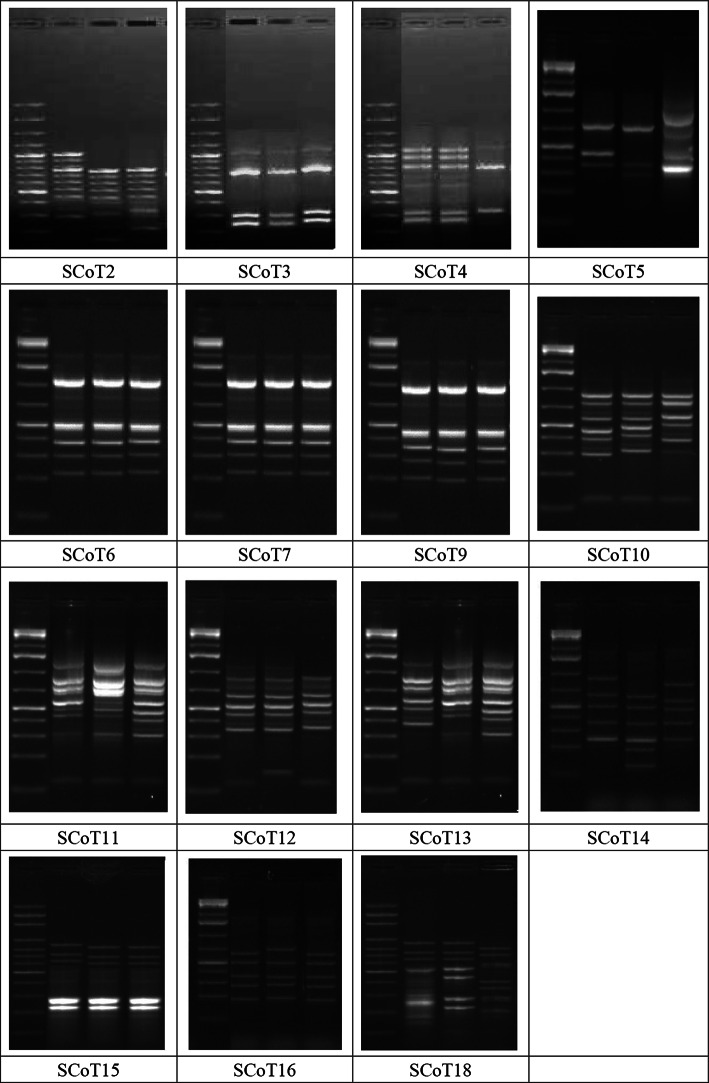
Profiles of fifteen SCoT markers for three *Moringa* Taxa. From left to right: Lane 1: DNA ladder (100 bp), Lane 2: *Moringa oleifera*, Lane 3: *Moringa peregrina*, and Lane 4: *Moringa stenopetala.* for more details about taxa, see Table 1.

Genetic analysis was conducted using 16 primers of start codon target (SCoT) markers to examine the molecular polymorphism among three *Moringa* taxa (see Table 1 for more details about taxa) (Fig. 6). The PCR reactions produced 112 amplicons with a length of 110–1341 bp, 18 of which were polymorphic, while 77 were monomorphic amplicons (Table 8). The polymorphism oscillated from no polymorphism by SCoT-14 to 80% by SCoT-05, with an average of 31%.

Unique amplicons summed up 17 bands plus 15 of which were negative unique bands. The marker SCoT2 registered the highest number of unique bands, while 3 were unique negative bands (SCoT11). This variance can be utilized to distinguish the studied markers revealed as unique bands.

Genetic-based identification of *Moringa* using SCoT markers enabled polymorphism detection to reveal the relationship between the studied taxa. Genetic characterization is crucial for managing the entire verification, classification, and genetic improvement^43^. The SCoT markers presented a high capacity for the determination of intra- and inter-genomic diversity because of their resultant polymorphism^59,86–88^. In this trend, some conclusions were obtained by Mahdy et al.^59^ on cowpea and El-Taher et al.^89^ on *Cichorium* taxa. The number of genetic parameters was estimated to evaluate the discriminatory power and diversity as detailed in Table 8. Heterozygosity (H) averaged 0.124 ± 0.02 and Shannon’s information index (I) averaged 0.18 ± 0.03. Notably, the SCoT-16, SCoT7, and SCoT9 recorded zero, and the SCoT-12 scored the highest value of 0.30 in both genetic estimations. The heterozygosity (H) is significant in polymorphism information content, which ranges from zero to one^90^. The derived data appeared to have a moderate informative value of diversity (Table 8). The Shannon index (I) is one of the most common measurements to estimate genetic diversity^91^. Previous studies are in general agreement with our results^59^. These genetic estimations could be used for determining diversity occurring between and within species and mirror heterozygosity in them. The heterozygosity is estimated based on the allele frequency.

**Table 8:** Polymorphism derived from fifteen SCoT primers.

Primer	MW	B	M	U	U+	U-	*P*	*P*%	I	H
SCoT2	255–1157	9	6	3	(1) 1157, 986 (3) 250	-	0	33.33	0.16	0.10
SCoT3	250–1144	6	3	2	(1) 980, 668	-	1	50.00	0.27	0.18
SCoT4	235–1147	7	4	2	(1) 668 (2) 510	(3) 986	1	42.86	0.23	0.16
SCoT5	275–630	5	1	1	(1) 370	(1) 275 (2) 630	3	80.00	0.50	0.35
SCoT6	218–1330	7	6	0	-	(2) 703	1	14.29	0.10	0.07
SCoT7	220–1341	7	7	0	-	-	0	0.00	0.00	0.00
SCoT9	245–1321	6	6	0	-	-	0	0.00	0.00	0.00
SCoT10	113–873	10	7	1	(3) 737	(3) 411	2	30.00	0.18	0.13
SCoT11	120–892	9	7	1	(3) 317	(2) 411	1	22.22	0.13	0.09
SCoT12	110–880	10	5	2	(1) 650 (3) 415	(3)880, 307, 275	3	50.00	0.30	0.21
SCoT13	125–870	10	6	1	(1) 740	(1) 555, 278 (2)317	3	40.00	0.25	0.18
SCoT14	125–647	7	4	2	(2) 125, 175	(2) 647	1	42.86	0.23	0.16
SCoT15	199–780	5	5	0	-	-	0	0.00	0.19	0.13
SCoT16	175–650	6	4	1	(2) 650	(2) 407	1	33.33	0.00	0.00
SCoT18	170–800	8	6	1	(3) 175	(3)800	1	25.00	0.14	0.10
Total	110–1341	112	77	17	17	15	18	31.25	0.182± 0.026	0.124± 0.018
Mean		8.13	5.13	1			1.2			

MW = molecular weight, B = number of bands, M = number of monomporphic band, U = number of unique bands, U + = number of positive unique bands, U-= number of negative unique bands, P = number of polymorphic bands, P%= polymorphism percentage, I (Shannon’s Information Index) = -1×(p×Ln(p) + q×Ln(q)); H (heterozygosity) = 2×p×q, where p is the frequency of the occurrence of amplicon, and q is null amplicon frequency.

Genetic correlation (Table 9) was summed up with the highest value of 0.798 between taxon 2 and taxon 3, followed by between taxon 1 and taxon 2 and between taxon 1 and taxon 3, 0.776 and 0.769, respectively. The genetic distance and variance were estimated for more classification (Table 9). The variance was recorded at 11.8, while the genetic distance oscillated from 2.69 to 2.92 with an average of 2.79. Genetic correlation revealed a very close genetic relationship among the studied taxa, and all dropped into the same class. That may be due to the variance decomposition targeting different genome loci by the SCoT technique^68,92^. Additionally, it may be due to the makeup of the taxon’s genome or the effect of evolution naturally.

**Table 9:** Genetic variance, similarity, and distance estimated between and within *Moringa* taxa under study.

Class	Value	1	2	3	Taxa
Within-taxa variance	11.667	1.000	0.776	0.769	**1**
Minimum genetic distance	2.687		1.000	0.798	**2**
Average genetic distance	2.787			1.000	**3**
Maximum genetic distance	2.92				

Noteworthy, *Moringa* species have economic uses in Egypt for their antioxidant, antimicrobial, and anti-diabetic activities. *Moringa* is traditionally used for treating wounds, fever, constipation, muscle pains, burns, labor pain, hypertension, malaria, stomach disorder, asthma, skin problems, and to expel a retained placenta^37^. Typically, taxonomic diversity refers to the genetic relationship between and within species. Three *Moringa* species were recorded in Egypt. Diversity measurements of *Moringa* were estimated as presented in Table 1. All *Moringa* taxa in the present study have at least one aspect of health benefit or environmental service. Health benefits include medicinal and pharmaceutical values, while environmental services are through shading, phytoremediation, and reducing soil loss. Despite the importance of *Moringa*, they are threatened by over-harvesting and over-cutting, climate change, and habitat loss.

For more diversity measurements, Dominance, Simpson diversity, Simpson reciprocal indices were calculated as presented in Table 10. Diversity is the variety and involves counting or listing species at its simplest level. Simpson index was estimated at 0.37, Dominance index was 0.63, and the Reciprocal index of Simpson was at 2.7. Simpson index for within species diversity was recorded at 0.145 (*M. stenopetala*), 0.28 (*M. peregrina*), and 0.855 (*M. oleifera*). *Moringa stenopetala* (6.875) scored the highest value of Reciprocal index, while the *Moringa oleifera* (0.852) recorded the highest value of Simpson. The results represented a high diversity for *M. oleifera* compared to others. The diversity index is a quantitative measure of the diversity of different organisms/species and how evenly the individuals are distributed among those species. The value of diversity index increases when the number of types increases and evenness increases^93^. Simpson index is weighted arthimetic average of proportional abundance and measures the probability that two individuals selected randomly from a sample will belong to the same species. This average will increase when the number of species decreases, and abundance of the most abundant species increases. Therefore, the Simpson index has small values in datasets of high diversity and vice versa. For this, Simpson index is usually expressed as a Dominance (its inverse) or reciprocal (its compliment) which is also called the Gini-Simpson index^93,94^. These results are in agreement with Shaltout and Bedair^95^. *Moringa* species are classified for conservation as: vulnerable (VU), very common (VC), and common (C). Propagation of *M. peregrina* had been successfully established in the Orman Botanic Garden and Shehab Mazhar Botanic Garden (private sector) in Giza^95^. A successful plantation of *M. peregrina* was done in South Sinai^96^. However, for a reason, *M. peregrina* was completely over-grazed in Orman Botanic Garden. Mortality of populations of *M. peregrina* is due to over-grazing^82^. Climate change impacts are becoming more evident, and mitigating its effects on localized species is a major challenge^95^.

**Table 10:** Simpson’s diversity and dominance indices of *Moringa* species in Egypt.

Diversity parameters	Moringa peregrina	Moringa oleifera	Moringa stenopetala	Total
# individuals	22	38	16	76
Simpson Index (D) = Σ n_*i*_(n_*i*_-1)/N(N-1)	0.280	0.852	0.145	0.370
Dominance Index = (1-D)	0.720	0.148	0.855	0.630
Simpson’s Reciprocal Index = 1/D	3.571	1.174	6.875	2.704

n_*i*_= number of individuals belonging to species _*i*_, N is the total number of individuals.

## Conclusion

Despite the important properties and potential uses of *Moringa* species among tropical plants, little research and development attention has been given to it. Besides its pharmaceutical use, *Moringa* is also a beneficial bio-material in farming. Several phytochemicals are yet to be identified in order to consider for all the potential therapeutic benefits. More attention is needed to *Moringa* as a priority plant to lessen malnutrition and as a source of farm income. This emphasizes the need for detailed and rigorous scientific study to assess the attributes of *Moringa* and utilize its full potential.

The obtained results reported that phenological features were assessed in the taxonomical association, which separate *M. peregrina* lonely (84%) and placed *M. stenopetala* with *M. oleifera* into a cluster score with a 95.3% similarity. A wide similarity has been observed between *M. oleifera* and *M. stenopetala* by the phytochemical compositions, especially the similarity in the major compounds such as 2-decenal, (E), 2-undecenal, and nonanal. Additionally, genetic estimations, i.e., heterozygosity (0.124), Shannon index (0.18), and Simpson index, confirmed the integration of these approaches.

Plant identification is the first step towards the determination of the basis for sustainable utilization of a taxon. Plant verification is based on the integration of micro- and macro-morphological assessment, molecular characterization, and phytochemical evaluation, which play a main role in the classification of within and between taxa. The morphology-based characterization is still the first step toward classification, genetic association, and changing climates. These distinct taxa and their phylogenetic associations were confirmed and evaluated in the current study using morphological, phytochemical, and molecular approaches. These methodologies proved helpful for classification, systematics, and phylogenetic studies at the taxonomical ranks of *Moringa.* We report that the leaf characteristics were the most important attributes in constructing the indented key for *Moringa* spp. We report that the genetic-based identification confirms the studies coming from morphological attributes. The integration of genetic and morphological along with phytochemical studies is helpful in the identification and verification of *Moringa* taxa. Finally, the genome assembly of *Moringa* among different countries diverges visibly with a firm amount of gene flow.

These taxa have the potential to be used as rich genetic resources for future studies in genetic diversity and germplasm preservation. The potential of these species can be benefited by; it would greatly benefit many countries that rely thoroughly on indigenous plants as a strength source for their maintenance. The rarity *Moringa* species, including *M. peregrina*, are also genetically vulnerable in their respective habitats due to several factors, ranging from unsustainable utilization, little scientific research and studies, and various threats. Further research is critically needed for *Moringa* species and should focus mainly on sustainable utilization and conservation. Efforts need to be expanded to pay the attention *Moringa* species rightfully deserve. If the genus *Moringa*, which consists of more potentially valuable species, can be further evaluated for its various characteristics, it can contribute to conservation and sustainable use. To sum up, the investigated *Moringa* species are a prolific source of bioactive secondary metabolites. There is an urgent need for more intensive bioprospecting studies and to establish a link between chemical composition and biological activities via molecular modeling experiments. Therefore, UPLC-QTOF-MS/MS metabolite profiling of the leaf extract is strongly recommended in future perspectives accompanied by docking studies.

## Data Availability

The taxa used are available for distribution to those interested in it. Requests for material should be mailed to National Gene Bank (NGB), Agricultural Research Center (ARC), 9 Gamaa St., PO Box: 12619; Giza, Egypt, or emailed to the corresponding authors: ehab.mahdy@arc.sci.eg, fatma.hamada@aswu.edu.eg.
